# Mitochondrial dynamics in vascular remodeling and target-organ damage

**DOI:** 10.3389/fcvm.2023.1067732

**Published:** 2023-02-13

**Authors:** Tong Zhu, Qingxun Hu, Yanggang Yuan, Huijuan Yao, Jian Zhang, Jia Qi

**Affiliations:** ^1^Department of Pharmacy, Xinhua Hospital Affiliated to Shanghai Jiao Tong University School of Medicine, Shanghai, China; ^2^Institute of Geriatrics (Shanghai University), Affiliated Nantong Hospital of Shanghai University, School of Medicine, Shanghai University, Shanghai, China; ^3^Shanghai Engineering Research Center of Organ Repair, School of Medicine, Shanghai University, Shanghai, China; ^4^Department of Nephrology, The First Hospital Affiliated to Nanjing Medical University, Nanjing, China

**Keywords:** mitochondrial dynamics, vascular remodeling, cardiovascular diseases, target-organ damage, fusion, fission

## Abstract

Vascular remodeling is the pathological basis for the development of many cardiovascular diseases. The mechanisms underlying endothelial cell dysfunction, smooth muscle cell phenotypic switching, fibroblast activation, and inflammatory macrophage differentiation during vascular remodeling remain elusive. Mitochondria are highly dynamic organelles. Recent studies showed that mitochondrial fusion and fission play crucial roles in vascular remodeling and that the delicate balance of fusion-fission may be more important than individual processes. In addition, vascular remodeling may also lead to target-organ damage by interfering with the blood supply to major body organs such as the heart, brain, and kidney. The protective effect of mitochondrial dynamics modulators on target-organs has been demonstrated in numerous studies, but whether they can be used for the treatment of related cardiovascular diseases needs to be verified in future clinical studies. Herein, we summarize recent advances regarding mitochondrial dynamics in multiple cells involved in vascular remodeling and associated target-organ damage.

## Graphical Abstract

The imbalance of mitochondrial fusion and fission plays an important role in vascular remodeling and target-organ damage. 
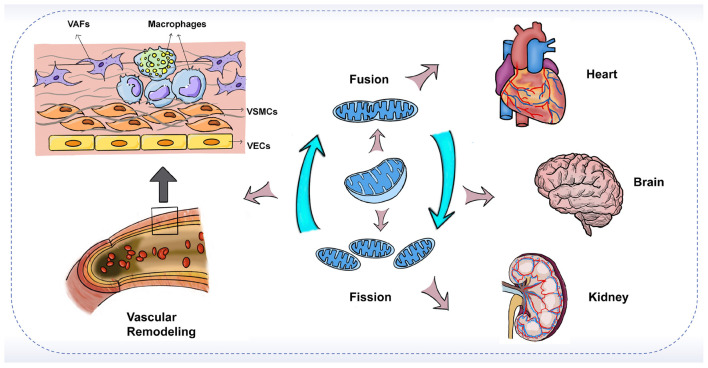

## Introduction

Cardiovascular disease (CVD) remains the leading cause of morbidity and mortality in developing countries, cardiovascular disease accounted for about 31 percent of global deaths in 2015 (1, 2). According to the statistics of the global burden of disease (GBD), from 2009 to 2019, the global deaths caused by CVD and vascular diseases were respectively related to ischemic heart disease (IHD, 16.17%), stroke (11.59%), cardiomyopathy and myocarditis (0.6%), other cardiovascular and circulatory diseases (0.49%), and chronic kidney disease (2.53%) [(IHME), (3)]. Vascular remodeling (VR) is an important process in various cardiovascular diseases. The vessel wall is an active and integrated organ composed of endothelial cells (ECs), smooth muscle cells (SMCs) as well as vascular fibroblasts (VAFs), and these cells can interact with each other (4). VR represents changes in vascular structure and arrangement, usually accompanied by activation of endothelial cells, migration and apoptosis of smooth muscle cells, degradation of the extravascular matrix (ECM), and disruption of the structural integrity of the vessel (4). Macrophages and inflammation also promote the development of vascular remodeling (5). Mitochondrial dysfunction is one of the important mechanisms mediating vascular remodeling and affects cellular homeostasis mainly by affecting cellular energy, reactive oxygen species (ROS) production, intracellular calcium levels, and apoptotic protein production (6). The vascular system is a tissue that does not require a lot of energy, thus the mitochondria here play the role of sensors, signaling hubs, and regulators of apoptosis, despite their role in energy metabolism (7, 8). In recent years, a growing number of studies have shown that defects in mitochondrial dynamics play a crucial role in the pathogenesis of several cardiovascular diseases (6). Mitochondrial dynamics is thought to have important functions in the growth, death, and migration of vascular endothelial and smooth muscle cells, and may also be involved in regulating the production or degradation of extracellular matrix, all of which contribute to vascular remodeling in cardiovascular diseases (9).

Mitochondria originated from a eukaryotic ancestor unified with an alpha-proteobacterium (10). Mitochondria have two relatively independent and functionally distinct membranes, the outer membrane (OM) and the inner membrane (IM) (11), which divide the mitochondria into two compartments. Located between the two mitochondrial membranes is the intermembrane space (IMS), and wrapped by the inner membrane are the matrix compartments (11). A small amount of DNA is present in the mitochondrial matrix, called mitochondrial DNA (mtDNA), which encodes a series of proteins critical to mitochondrial respiration (12). Despite the long evolutionary history before being perceived as eukaryotic organelles, the important role of mitochondria in eukaryotic cell function is deeply ingrained (13). As is known to all that mitochondria are the “powerhouses” of cells, responsible for producing most of the ATP in cells through oxidative phosphorylation (OXPHOS). In addition, mitochondrial electron transport chains are also vital to the regulation of mitochondrial calcium (14). Mitochondria can be the main source of ROS during electron transport to produce ATP, which can both promote cell death and act as signaling molecules. Therefore, mitochondria are also key regulators of cell death (15). To achieve these functions, mitochondria have become organelles that exhibit remarkable dynamics (16). Mitochondria regulate their morphology and control their number and size through fusion and fission, a continuous cycle of fission and fusion is also known as mitochondrial dynamics (17). Mitochondrial dynamics is closely related to mitochondrial function. The balanced fusion and fission events of mitochondria maintain the health of mitochondria and their host cells and organisms (15, 17). There is evidence that an imbalance of mitochondrial dynamics plays a crucial role in the pathogenesis of many cardiovascular diseases (6). This review summarizes recent progress in the understanding of the role of mitochondrial dynamics in vascular remodeling and related target-organ damage.

## Mitochondrial fusion and fission

### Mitochondrial fusion

**FIGURE 1:**
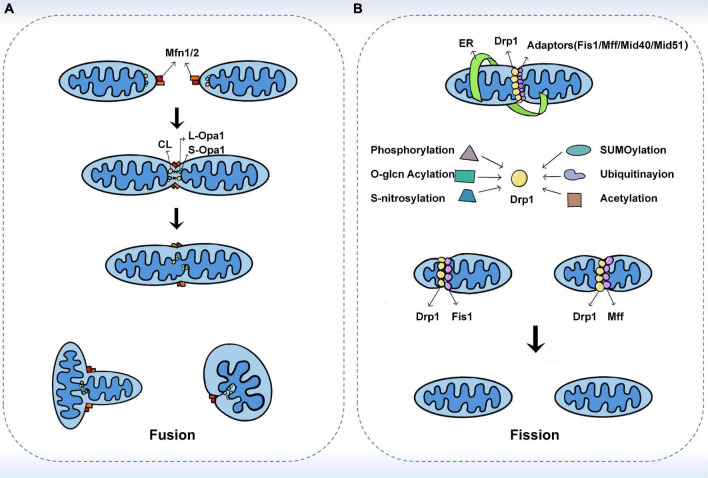
The fusion and fission machinery. **(A)** The mechanisms of mitochondrial fusion. Mfn1/2 mediate outer membrane fusion. Opa1 mediates inner membrane fusion and maintains cristae structure. Under stress, L-Opa1 is activated and S-Opa1 can regulate the fusion activity of L-Opa1. **(B)** The mechanisms of mitochondrial fission. Drp1 plays a central role in mitochondrial fission. Several kinds of post-translational modifications of Drp1 may affect the recruitment of Drp1 from the cytoplasm. The adaptors including Fis1, Mff, MiD49, and MiD51 also have substantial roles in the recruitment of Drp1 to mitochondria.

Mitochondrial fusion refers to the integration of the outer and inner membranes of two adjacent mitochondria into one, resulting in a fibrous extension and network structure of mitochondria (18). Mitochondrial fusion has been shown to occur through a sequential event of the outer mitochondrial membrane (OMM) and inner mitochondrial membrane (IMM) fusion, with Mitofusins (Mfns) and Optic atrophy protein 1 (Opa1) mediating the fusion of the outer and inner membranes, respectively (19, 20). In a typical mitochondrial fusion reaction, the endpoints of two mitochondria collide, with the outer membrane fusing first and then the inner membrane fusing at the collision site. Fusion events can also occur between ends and sides, or within a single mitochondrion to form a ring structure (16). As a result of fusion, the content is mixed and the matrix is exchanged, but mtDNA exchange is limited despite being in the matrix (21). In addition to complete fusion, mitochondrial fusion can take different forms, such as instantaneous fusion, the so-called “kiss-and-run” encounter, in which no obvious merger or structural rearrangement occurs (22) (Figure 1A).

### Regulators of mitochondrial fusion

Mitochondria primarily mediate fusion through three membrane GTPases of the dynamin-related protein (DRP) superfamily, including Mitofusin 1 (Mfn1), Mitofusin 2 (Mfn2), and Optic atrophy protein 1 (Opa1). Among them, Mfn1/2 is located on the outer mitochondrial membrane and its main function is to facilitate mitochondrial docking and fusion (18). The mitochondrial fusion mechanism of Mfns has been investigated based on its topology (23). In the past, Mfns were thought to be inserted into the OMM through two transmembrane structural domains (TMs). TMs are separated by a short loop containing GTPase and coil-coil heptad repeat 1 (HR1) structural domain at the N-terminal end, while the C-terminal end carries the HR2 structural domain (24, 25). It has been proposed that Mfns establish a linkage between paralogous mitochondria *via* dimeric antiparallel *trans* interactions at their HR2 structural domains, followed by GTP hydrolysis to produce an OMM fusion (23). However, a recent study showed that only one transmembrane structural domain exists in human Mfns, which places the N-terminal GTPase and HR1 structural domains in the cytoplasm and the C-terminal HR2 structural domain in the mitochondrial membrane gap (26). Several studies have proposed that the bolting of the two OMMs occurs through oligomerization of the GTPase structural domains in the Mfns. Upon GTP binding and hydrolysis, the GTPase structural domain undergoes a conformational change leading to its oligomerization, which facilitates the docking and subsequent fusion of the two mitochondria at the two outer membranes (27, 28). Mattie et al. demonstrated that with increasing levels of oxidized glutathione, the two cysteine residues located in the HR2 structural domain can be oxidized, leading to the formation of disulfide bonds between Mfn molecules and then oligomerization occurs (26). This new mechanism suggests that redox signaling plays a crucial role in OMM fusion. Furthermore, studies in cells have shown that Mfn1 and Mfn2 have different functions. For example, Mfn1 has a higher GTPase activity than Mfn2 and therefore promotes mitochondrial fusion more efficiently (29). Opa1-mediated fusion is also dependent on Mfn1 rather than Mfn2 (30). This explains that overexpression of either Mfns leads to perinuclear mitochondrial aggregation but with different results. Mfn1 induces mitochondrial fragmentation, while Mfn2 exhibits swollen spherical mitochondria (31).

Mitochondrial endosomal fusion is mediated by the direct action of optic atrophy 1 (Opa1) and cardiolipin (CL), a specific component localized to the inner mitochondrial membrane (24). Unlike Mfns, only one of the two opposing mitochondria needs to carry Opa1 for endosomal fusion to occur (16). Ban et al. showed that Opa1 mediates endosomal fusion *via* heterotrimeric Opa1-cardiolipin interactions. Similarly, when cardiolipin was not present, membrane fusion was not observed even when L-Opa1 was present on both sides of the membrane (32). The encoding of Opa1 forms eight isoforms during transcription by differential splicing in exons 4, 4b, and 5b. All isoforms contain an N-terminal mitochondrial targeting sequence (MTS) and a generic exon 5 encoding the S1 protein hydrolysis site (33). The MTS is removed by matrix processing protease (MPP) during N-terminal entry into the matrix, while the retention or not of the S1 site determines the isoforms of Opa1 (34). If the S1 site remains intact, a long isoform of Opa1 (L-Opa1) is produced that is anchored to the endosomal membrane. In the steady state, approximately half of Opa1 exists as L-Opa1. In contrast, if the S1 site is cleaved by the Oma1 protease, short isoforms of Opa1 (S-Opa1) are produced, and they form a complex through the L-Opa1 isoform to regulate fusion activity. It was shown that the addition of S-Opa1 enhanced the membrane fusion activity of L-Opa1 *in vitro* (32, 34). Alternatively, variant S-Opa1 can be produced by the shearing of S2 proteins by the Yme1L protease (34, 35). Previous and recent studies have shown that fusion requires the co-action of long and short isoforms under basal conditions (36, 37). However, several different findings also exist. For instance, some studies have shown that L-Opa1 alone promotes IMM fusion (34, 38). Moreover, S-Opa1 has been proposed to promote fission (39).

### Mitochondrial fission

Mitochondrial fission is the division of one mitochondrion into two smaller mitochondria, usually asymmetrically, resulting in the formation of two unequal-sized daughter mitochondria (16, 40). The core reaction of mitochondrial division is the contraction and rupture of the two mitochondrial membranes, which is driven by the recruitment of Drp1 to the mitochondria *via* its receptor protein located on the outer membrane and is regulated at multiple levels (40–42). During the life cycle of mitochondria, fission can both produce new mitochondria and eliminate dysfunctional mitochondria through mitochondrial phagosomes (43, 44). Previous studies have shown that fission is the basis of mitochondrial proliferation and degradation (43). However, recent studies have found that the regulation of organelle levels, that is, the localization of fission sites, may be a key morphological feature leading to different mitochondrial fates (45). Super-resolution microscopy was used to analyze the mitochondrial division and then two spatially different types of division were defined in this study. Midzone division occurs at the center of the organelle (within the central 50%), whereas peripheral division occurs at both ends of the mitochondria (less than 25% from the tip). Division in the outer region contributes to the shedding of damaged material into smaller mitochondria for mitochondrial phagocytosis, while division in the middle region causes the mitochondrial proliferation (45) (Figure 1B).

### Regulators of mitochondrial fission

The central reaction of mitochondrial division is the contraction and rupture of two mitochondrial membranes (46). Recently, Kraus et al. introduced the concept of “mitochondrial divisome.” That is, in eukaryotic cells, the core components of the division include dynamin-related protein 1 (Drp1) and its adapters, while other organelles such as the endoplasmic reticulum (ER), actin, contact sites between organelles, and specific lipids on the outer membrane are classified as accessory components, which together regulate and control mitochondrial division (47).

Drp1 is a cytoplasmic protein that acts as a key GTPase in mitochondrial fission (18). Once recruited from the cytoplasmic pool to the outer mitochondrial membrane, it forms helical oligomers that induce membrane contraction and fracture. In contrast, the deletion of Drp1 blocks mitochondrial division, causing mitochondria to lengthen or expand (40). Whether Drp1 can independently mediate mitochondrial division has not been fully demonstrated (48, 49). It has been suggested that Dynamin 2 (DNM2) is also recruited to the mitochondrial division site and is involved in the final membrane break event, and that knockdown of DNM2 inhibits mitochondrial fission, revealing its functional importance in fission (48). However, the necessity of DNM2 in mitochondrial fission has been challenged by many new studies. Drp1 itself has been shown to sever membrane tubules (50). Moreover, the loss of all conventional kinesins, including DNM1, DNM2, and DNM3, did not lead to mitochondrial inhibition of fission (51). These suggest that Drp1 can independently lead to mitochondrial fission. Nonetheless, DMN2 was also observed at the mitochondrial fission site on both sides of Drp1 (48), so it may play a non-essential role in fission. Post-translational modifications, such as phosphorylation, further regulate Drp1-mediated mitochondrial fission (52). MAPK1 can stimulate the phosphorylation of Ser616, which enhances Drp1 activity and leads to increased mitochondrial fission. On the other hand, PKA-induced phosphorylation of Ser637 inhibits Drp1 GTPase activity and fission, whereas Ca^2+^-activated dephosphorylation triggers fission (18). Recently, the mitochondrial phosphatase PGAM5 has also been found to be involved in Ser637 dephosphorylation, the loss of which impairs mitochondrial fission and increases cellular senescence (53). In addition, o-glcn acylation, SUMOylation, s-nitrosylation, and ubiquitination can also regulate Drp1 activity (18). O-glcn acylation and s-nitrosylation of Drp1 increase its fission activity (54, 55), while mitochondria-associated protein ligase (MAPL)-mediated SUMOylation contributes to the stabilization of Drp1 function (56). Parkin can degrade the mitochondrial splitting protein Drp1 by ubiquitination (57). Apart from the above, recent studies have shown that excess lipid supply can create an intracellular environment in mice that promotes Drp1 acetylation, which in turn increases its activity and mitochondrial translocation, leading to cardiomyocyte dysfunction and death (58). It has been reported that low doses of methylmercury (MeHg) induced depolymerization of Drp1 at Cys624-S(n)H, leading to filamin-dependent activation of Drp1 and mitochondrial hyperfission, which increased cardiac fragility to mechanical load. In contrast, cilnidipine, a blocker of Drp1 and filamin-A interaction, inhibited mitochondrial hyperfission in neonatal rat cardiomyocytes induced by low-dose MeHg exposure (59).

Adapters for Drp1 include four extramembrane proteins, Fis1, Mff, MiD49, and MiD51. Each adapter can independently recruit Drp1 into mitochondria, and there are also indications of interactions between MiDs and Mff (60, 61). In yeast, Fis1 plays a central role in mitochondrial fission, but in mammals, cells lacking Fis1 show little or no fission deficiency (41). Mff, MiD49, and MiD51 appear to have a more important role in mammalian Drp1 recruitment. Mff deficiency results in a reduced recruitment of Drp1 to mitochondria and disrupts fission, whereas Mff overexpression enhances mitochondrial fragmentation (61). MiDs are more complex, as their low protein expressions lead to enhanced mitochondrial fission, but overexpression leads to significant mitochondrial elongation (62). However, a recent study by Kleele et al. showed that there is more than one type of mitochondrial division and that Fis1 and Mff play different roles in the different forms of mitochondrial division (45). When mitochondria are damaged or stressed, Fis1-mediated peripheral division is increased, predicting mitochondrial autophagy. In contrast, Mff has no role in the peripheral division but plays a role in the midzone division. Mid-region division is usually increased in situations that stimulate cell proliferation (45). This study also raises exciting questions, such as whether other factors are specifically involved in a peripheral division or midzone division. In this regard, MiD51 and MiD49 are of particular interest because this new study does not provide conclusive results regarding their role.

In the complex environment of living cells, accessory components of the “mitochondrial divisome,” such as the endoplasmic reticulum (ER) and lysosomes, as well as actin, cytoskeleton-related binding proteins inverted formin 2 (INF2) and Spire1C, which are required for initiation and control of mitochondrial fission (47). The contact site between mitochondria and the ER is important for coordination between the two organelles and may be the site where the first step of the division process occurs (63). Before Drp1 is recruited, ER-bound INF2 cooperates with mitochondria-anchored Spire1C to regulate mitochondrial contraction to a size that allows Drp1-oligomeric loop formation at the contact site between the two organelles (64). This process may also be related to the motor protein myosin II (65). However, it has also shown that Ca^2+^-dependent inner membrane (IMM) contraction represents the initiating event of mitochondrial fission (66). IMM contraction occurs before outer membrane contraction and is regulated by Opa1, a kinetic protein that controls mitochondrial fusion. Opa1 interacts with the MICOS complex that connects the outer and inner membranes and promotes inner membrane contraction (66). Interestingly, endosome contraction is enhanced in the absence of Drp1, suggesting that cells can compensate for the absence of mitochondrial division by increasing endosome contraction (67).

Contact of lysosomes and *trans*-Golgi networks with mitochondria is also associated with fission. Lysosomes bind at the site of mitochondrial contraction before fission (68). The mitochondria-localized GTPase-activating protein TBC1D15 is involved in fission along with the lysosome-associated RAB7. Recently, phosphatidylinositol 4-phosphate [PI(4)P] *trans*-Golgi vesicles and ARF1 were discovered to co-localize at Drp1-positive mitochondrial fission sites, which are also labeled by lysosomes (69). The deletion of ARF1 or PI(4)P increased mitochondrial length but not Drp1 recruitment, suggesting that loss of PI(4)P delayed the late phase of Drp1-mediated fission.

Mitochondrial lipids are also involved in the regulation of mitochondrial fission and have different roles. In addition to its role in fusion, cardiolipin binds to Drp1, drives Drp1 oligomerization, and stimulates its GTPase activity (70). In contrast, phosphatidic acid interacts with Drp1 and inhibits its function (71). The mitochondrial phospholipase MitoPLD can act as a key regulator in the conversion of stimulatory cardiolipin and inhibitory phosphatidic acid. Interestingly, Drp1 can form a complex with DMitoPLD to generate phosphatidic acid from cardiolipin (72). It will be exciting to further decipher how the interactions of Drp1, the actin cytoskeleton, phospholipids, and other organelles coordinate to control mitochondrial division.

### Balance of fusion and fission

Nearly 100 years ago, mitochondrial motility and fission were first observed with light microscopy (73), and by the 1980s and 1990s, the study of mitochondrial dynamics continued to advance with the development of technology. First with phase-contrast microscopy, then with vital dyes, and finally with targeted fluorescent proteins, it was confirmed that mitochondria are in a constant process of division and fusion (74–76). The fact that mitochondrial fission and fusion are considered to be critical processes for mitochondrial and cellular health has not been fully appreciated until recent years (46, 77). Fission is important to ensure the number and distribution of mitochondria in daughter cells, and fusion allows content exchange between fused mitochondria to ensure optimal mitochondrial activity (18). Mitochondrial fusion and fusion usually occur simultaneously in a balanced manner within the cell, and the net balance between fusion and fission controls the size, number, shape, and activity of mitochondria (40). Disruption of homeostasis leads to imbalances in mitochondrial function, ultimately leading to a variety of diseases, including cardiovascular disease, neurodegenerative diseases, and cancer (78). An increase in fission or a decrease in fusion will result in more short or small mitochondria. In some cells, the shape itself has important functional consequences. In neurons, for example, the transport of small mitochondria to nerve terminals may be more efficient compared to long mitochondria (16). In addition, the size of mitochondria is crucial for their degradation by mitochondrial phagocytosis (41). However, the proper balance of fusion and fission seems to be more important compared to the absolute level of each process. For example, mice lacking Mff show severely reduced mitochondrial fission, significantly reduced OXPHOS activity in cardiomyocytes, and extensive fibrosis in cardiac tissue. In contrast, these defects can be completely rescued by concomitant loss of Mfn1 (79). A new study shows that mitochondrial elongation factors 1 and 2 (MIEF1/2) regulate mitochondrial fusion through direct interaction with the fusion proteins Mfn1 and Mfn2, in addition to their role in the fission machinery. MIEF1/2 competitively reduces the interaction of hFis1 with Mfn1 and Mfn2, alleviating hFis1-induced mitochondrial fragmentation and contributing to mitochondrial fusion (80). Furthermore, there is evidence that cellular feedback mechanisms can rebalance mitochondrial dynamics in part through compensatory responses when fission is disturbed. For instance, when Drp1 was deleted, the levels of Mfn1 and Mfn2 decreased by approximately 50% (81, 82). Based on the above, it can be argued that the delicate balance between mitochondrial fusion and fission plays an integral role in maintaining the mitochondria functions and determining cell fate. In fact, the reciprocal regulation of fission and fusion is thought to be an emerging trend in the remodeling of mitochondrial networks, where multiple regulatory pathways influence both processes (83).

## Mitochondrial dynamics and mechanism of vascular remodeling

**FIGURE 2:**
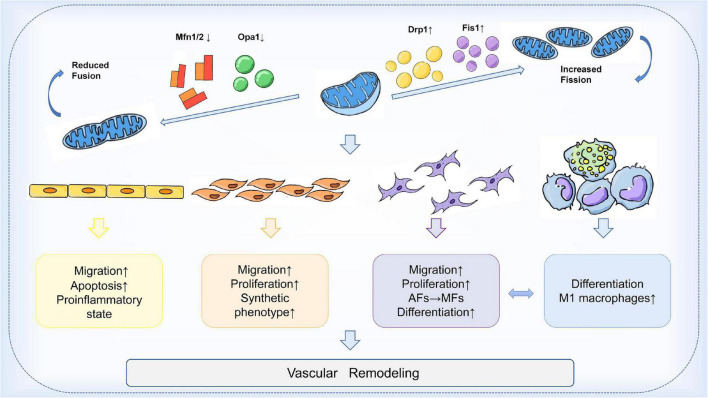
Mechanism of mitochondrial dynamics in vascular remodeling. During vascular remodeling, multiple cells of the vessel wall are regulated by mitochondrial dynamics. Mitochondrial division and fusion play an important role in regulating the migration, apoptosis, and pro-inflammatory state of endothelial cells, and are also involved in the transformation of the synthetic phenotype of VSMCs and the process of phenotypic changes from adventitial fibroblasts (AFs) to myofibroblasts (MFs). Mitochondrial kinetic imbalance is also an important factor in the proliferation and migration of VSMCs and AFs. In addition to these, the delicate balance between mitochondrial fission and fusion seems to be involved in regulating macrophages and the interactions between VSMCs and macrophages, thus affecting vascular remodeling.

In the arterial wall, endothelial cells (ECs), vascular smooth muscle cells (VSMCs), fibroblasts (VAFs), and macrophages are all important players in the process of vascular remodeling (VR). Mitochondrial dynamics are essential for the proper function of all key cell types involved in the development of vascular remodeling. Endothelial dysfunction is often considered a marker of atherosclerosis (84). Inhibition of Drp1 in apolipoprotein E (ApoE) knockout diabetic mice reduces endothelial dysfunction and atherosclerosis (85). Mfn2 expression is reduced in atherosclerosis models, and conversely, Mfn2 overexpression reduces atherosclerotic lesions in rabbits (86, 87). In addition, endothelial cells from patients with reduced vascular function exhibit increased expression of Fis1 and mitochondrial fragments, a phenomenon that can be rescued by *in vitro* fission inhibition (88). On the other hand, vascular smooth muscle cells (VSMCs) also proliferate and migrate during vascular reconstitution. Reduction of Mfn2 is associated with the activation of VSMCs by platelet-derived growth factor (PDGF), while inhibition of fission attenuates VSMCs proliferation (89). In an *in vitro* aortic ring assay, inhibition of Drp1 significantly reduced the proliferation and migration of VSMCs and decreased neovascularization in a rat carotid balloon injury model (90). Furthermore, macrophage-mediated inflammation is thought to accelerate the process of vascular remodeling. Blocking Drp1 reduces the accumulation of inflammatory macrophages in injured vessels and inhibits the growth and migration of VSMCs (91). In conclusion, these findings suggest that the expression of mitochondrial dynamin proteins plays an integral role in the development of vascular remodeling. Modulation of the balance of mitochondrial fission and fusion is expected to be an effective therapeutic strategy to slow the progression of vascular remodeling (Figure 2).

### Vascular endothelial cells (ECs)

All arterial vessels from the heart to the capillaries have a continuous layer of endothelial cells (ECs), which constitute the innermost layer of the vessel wall and are in direct contact with blood flow (92). ECs provide an impermeable barrier between blood and tissues, ready to sense the environment and signal the regulation of vascular function. Therefore, alterations in endothelial function are often considered to be the beginning of vascular remodeling (92). On the other hand, mediators released by ECs can modulate the function of VSMCs and thus exert a decisive influence on vascular remodeling (93). Endothelial cells derive most of their energy from anaerobic glycolysis, and ATP production from oxidative phosphorylation reactions represents only a small fraction of the total energy they obtain. Compared to energy production, endothelial cell mitochondria seem to play a more dominant role in cellular homeostasis and activation of signaling cascades (94, 95). In addition to the traditional functions of regulating ROS, maintaining Ca^2+^ concentration, and integrating apoptotic stimuli, intact mitochondrial dynamics have an important role in regulating apoptosis, angiogenesis, and the pro-inflammatory state of the endothelium (96).

A series of studies have confirmed the involvement of the imbalance of mitochondrial fusion and division in the regulation of endothelial cell function. Lugus et al. demonstrated that Mitofusins have an important role in endothelial cell survival and angiogenic function (96). In VEGFA-induced human umbilical vein endothelial cells (HUVECs), knockdown of any of the Mfns resulted in disruption of the mitochondrial network and reduction of the mitochondrial membrane potential (96). At the same time, the ablation of Mfns impaired endothelial cell viability and increased apoptosis. Knockdown of Mfn2 alone results in reduced reactive oxygen species production, whereas knockdown of Mfn1 alone selectively reduces VEGF-stimulated Akt-eNOS signaling (96). Thus, Mfn1 and Mfn2 regulate endothelial cell migration, differentiation, and survival by affecting different aspects of angiogenic signaling. A recent study also showed that reduced Mfn2 expression leads to the uncoupling of endoplasmic reticulum-mitochondrial contacts, which in turn leads to enhanced mitochondrial fission, mitochondrial fragmentation, and as a result, increased ECs death (97). Several other studies in recent years have also shown an important role of mitochondrial fission and fusion in endothelial cell angiogenesis (98, 99). By activating pyruvate kinase myozyme 2 (PKM2), glycolytic and mitochondrial fusion events are significantly enhanced, thereby promoting angiogenesis in vascular-resident endothelial progenitor cells (VR-EPCs). Conversely, inhibition of PKM2 activity leads to a decrease in glycolysis and a significant promotion of mitochondrial fission, resulting in reduced expression of angiogenesis-related genes (98). Another study showed that increased phosphorylation of Drp1 in HUVECs enhances its activity in regulating mitochondrial fission and induces endothelial cell migration through the AMPK signaling pathway (99). The same results were obtained in a mouse model.

An increasing number of studies have found morphological alterations, loss of mitochondrial networks, and increased expression of fission proteins in mitochondria in endothelial cells under diabetic conditions. Makino et al. found in *in vitro* experiments that decreased levels of Drp1 in high-glucose (HG)-treated mouse coronary artery endothelial cells (MCECs) led to increased mitochondrial elongation, while increased levels of Drp1 may lead to mitochondrial fracture. This increases mitochondrial fragmentation and exhibits disruption of normal endothelial cell functions (100). In this study, similar results were obtained in animal experiments. Levels of Opa1 were significantly reduced in MCECs from diabetic mice, and levels of Drp1 were significantly increased, ultimately leading to an increase in mitochondrial fission (100). However, alterations in the levels of other fusion or fission-related proteins, such as Mfn1/2 and Fis1, were not confirmed in the present study. Similarly, Trudeau et al. discovered increased mitochondrial fragmentation in rat retinal endothelial cells cultured in an HG medium, which may underlie retinal endothelial cell apoptosis in diabetic retinopathy (101). A recent study showed that mitochondrial fission genes Fis1 and Drp1 were overexpressed under HG conditions, and that downregulation of Fis1 and Drp1 preserved the normal morphology of mitochondria, inhibited mitochondrial fission, and reduced apoptosis in retinal endothelial cells (102). Emerging evidence suggests that altered mitochondrial fusion-fission homeostasis is divided into a compensatory phase, an equilibrium shift phase, and a loss of compensatory phase (103). Initially, Mfn1 and Mfn2 exhibit a significant decrease while Drp1 and Fis1 are elevated, and mitochondrial fusion/fission homeostasis is disturbed, but normal mitochondrial membrane potential (MMP) can still be maintained. Then, with prolonged high glucose exposure, mitochondrial fusion/fission imbalance and mitochondrial fragmentation occurred. Finally, Opa1 expression decreased and mitochondrial fragmentation triggered endothelial cell apoptosis (103). Notably, Fis1 expression initially increased and decreased at the end, so Fis1 may be a predictor of changes in mitochondrial fusion-fission homeostasis during vascular injury. Both *in vitro* and *in vivo* results consistently showed that altered mitochondrial fusion-fission homeostasis could trigger high-glucose-induced vascular endothelial injury. The mechanism responsible for this outcome may be related to the AMPK signaling pathway.

The pro-inflammatory state associated with mitochondrial fission is also a factor contributing to vascular endothelial cell injury. Drp1-mediated mitochondrial fission plays a fundamental role in the regulation of inflammation (6). For example, mRNA transcript levels of inflammatory factors such as IL-1β, IFNγ, and TNFα are higher in Drp1-deficient mice (104). A recent study identified a unifying mechanism linking impaired mitochondrial dynamics to endothelial inflammation. The activity of Drp1 was shown to be necessary for the induction of inflammation by the proinflammatory proteome in endothelial cells. Drp1 and its receptor Mff are involved in mediating endothelial Inflammatory NF-κB activation and vascular cell adhesion molecule-1 (VCAM-1) induction (104). In cultured endothelial cells, inhibition of Drp1 activity or expression inhibits not only mitochondrial fission but also NF-κB activation, VCAM-1 induction, and leukocyte adhesion induced by these proinflammatory factors. On the other hand, attenuation of inflammatory leukocyte adhesion was also observed in Drp1 heterozygous-deficient mice and endothelial-type Drp1-silenced mice. Interestingly, this study also demonstrated that inhibition of the typical NF-κB signaling pathway could also in turn inhibit endothelial cell and mitochondrial fission. Thus, there may be interdependence between the typical NF-κB cascade activation and mitochondrial fission pathways in endothelial cells, which together regulate endothelial cell inflammation. In addition, it was shown that the classical anti-inflammatory drug salicylate could also maintain mitochondrial fission-fusion homeostasis by inhibiting NF-κB.

Although the mitochondrial content of vascular endothelial cells is relatively low, mitochondria are a key site of ROS production (105). Chronic accumulation of mitochondria-derived ROS (mROS) contributes to vascular fibrosis and remodeling secondary to endothelial cell apoptosis (106). mROS-induced oxidative stress accelerates endothelial cell senescence, particularly by impairing the endothelial cell migration response, reducing paracrine capacity, and increasing endothelial permeability. These characteristic alterations impede endothelial regeneration and angiogenesis (105). Moreover, disruption of mitochondrial redox homeostasis in ECs can cause chronic inflammation, which in turn disrupts vascular endothelial homeostasis (107). Mitochondrial fusion and fission are directly or indirectly associated with mitochondrial energy metabolism (108). When Drp1 activity is inhibited, mitochondrial ROS production and ATP levels are subsequently reduced (109). Studies have consistently shown that mitochondrial respiration and energetics are also inhibited in cardiac-specific Drp1-KO mice (110). It was shown that silencing of Fis1 or Drp1 under high glucose conditions may maintain endothelial NOs (eNOs) activity and NO bioavailability by decreasing mitochondrial ROS (88). Overall, fusion-fission imbalance in endothelial mitochondria can lead to loss of mitochondrial function and increased ROS production, while excessive accumulation of ROS in turn further disrupts normal mitochondrial function and morphology. This vicious cycle promotes the initiation of vascular remodeling (111).

### Vascular smooth muscle cells (VSMCs)

Vascular smooth muscle cells (VSMCs) are key components of the medial layer of arteries and major determinants of vascular tone (112). VSMCs have significant phenotypic plasticity and can respond to functional demands and external stimuli by transitioning from a contractile to a synthetic phenotype (113). In the contractile state, VSMCs, together with ECs, regulate vascular homeostasis by controlling blood flow. On the other hand, in response to vascular injury, VSMCs lose contractile markers and switch to a synthetic phenotype, becoming dedifferentiated and with enhanced proliferation and migration capacity (114, 115). Phenotypically modified VSMCs play important roles in vascular remodeling (116). Recent findings suggest that the phenotype switching of VSMCs is associated with dysfunctional mitochondrial dynamics (117). Notably, VSMCs are energy-dense cells that are susceptible to organelle dysfunction. Thus, mitochondrial dysfunction caused by fusion-fission imbalance may also aggravate vascular remodeling by causing the calcification of VSMCs (118).

In previous studies, mitochondrial fission has been shown to have a crucial role in pulmonary artery smooth muscle cells (PASMCs) hyperproliferation (119). In addition, VSMCs exposed to platelet-derived growth factor-BB (PDGF) showed mitochondrial fragmentation and a significant decrease in Mfn2, accompanied by a decrease in glucose oxidation and an increase in fatty acid oxidation (89). In contrast, Mdivi-1 inhibited Drp1-mediated mitochondrial division and exhibited anti-VSMCs proliferation and migration activity in both cellular and animal experiments (89). These results suggest that changes in mitochondrial morphology and energetics underlie the overproliferation of the VSMCs synthetic phenotype. A recent study has shown that astaxanthin (ATX) can inhibit mitochondrial fission and thus VSMCs proliferation by reducing Drp1 phosphorylation, exhibiting potential for therapeutic vascular remodeling (120). Mitochondrial fusion has also been shown to be associated with VSMCs proliferation. Glucagon-like peptide-1 (GLP-1) stimulates mitochondrial fusion through the PKA/Drp1 signaling pathway, increasing mitochondrial activity and reducing VSMCs dedifferentiation (121). *In vitro* and *in vivo* experiments suggest that GLP-1 can inhibit vascular remodeling through a mitochondrial dynamics-dependent pathway. In addition, Mfn2 is an important inhibitor of VSMC proliferation (122). Studies showed that in VSMCs, the specific site of Mfn2 could be phosphorylated by PKA, leading to an inhibitory effect on the proliferation of VSMCs. Also, an inhibitory effect on endothelial proliferation and restenosis was demonstrated in a rat balloon injury model. Interestingly, although PKA-specific phosphorylation plays a key role in Mfn2-mediated VSMCs growth inhibition, it is not related to its role in the regulation of mitochondrial morphology. It can be considered a non-canonical action of mitochondrial dynamics proteins (123). Another study showed that overexpression of Mfn2 triggered vascular smooth muscle apoptosis, whereas silencing Mfn2 prevented vascular smooth muscle apoptosis (124). Similarly, this is an independent way of mitochondrial fusion.

Altered mitochondrial dynamics also have important influences on PDGF-induced VSMCs migration, which leads to VSMCs migration by regulating calmodulin-dependent kinase II (CaMKII) (114). Indeed, altered mitochondrial morphology leading to metabolic dysfunction, damage to mitochondrial components, and mitochondria-mediated ROS generation are also mediators involved in VSMCs proliferation and migration, a potential mechanism promoting cardiovascular disease (112). A related study showed that PDGF induces mitochondrial shortening *via* DLP1 in VSMCs in mice and that perturbing mitochondrial fission by silencing Drp1 significantly reduces VSMCs migration (125). This suggests that mitochondrial fission is an indispensable process for VSMCs migration. In DLP1-K38A transgenic mice, mitochondrial fission was reduced *in vivo*, leading to a restricted development of endothelial proliferation induced by arterial injury. This study demonstrates that inhibition of mitochondrial fission results in a significant reduction in PDGF-induced VSMCs migration and line injury-induced intimal hyperplasia. Moreover, the correlation between altered mitochondrial morphology, such as mitochondrial shortening, and cell migration was verified in this study. Recent findings show that Krüppel-like factor 5 (Klf5), a transcription factor important for vascular remodeling, was shown to be associated with mitochondrial fission. knockdown of Klf5 was enhanced, while Klf5 overexpression inhibited mitochondrial fission (126). Furthermore, activation of the p38 MAPK pathway can promote VSMCs proliferation and arteriogenesis *in vitro* in response to Mfns through PGC1α-dependent mitochondrial dynamics (127). Overall, the above findings suggest that mitochondrial dynamics plays a key role in VSMCs proliferation and migration.

Vascular smooth muscle cells phenotypic switching and its driven calcification process play a key role in regulating vascular wall remodeling. As the studies were explored in depth, the process of calcification exhibiting mitochondrial dysfunction was gradually exposed (115, 118). An excessive increase in mitochondrial fission was observed in VSMCs treated with phosphate and a similar disruption of mitochondrial structural integrity was found in a rat model of adenine-induced aortic calcification (128). Drp1 is involved in the transformation of VSMCs into an osteoblast-like phenotype by regulating mitochondrial fission. Increased mitochondrial fragmentation and enhanced Drp1 expression can be observed during calcification events (128), while further studies have shown that inhibition of Drp1 attenuates cardiovascular calcification under some degree of oxidative stress (129). It has been confirmed that melatonin can attenuate β-glycerophosphate (β-GP)-induced VSMCs calcification *via* the AMPK/Drp1 signaling pathway. In this process, melatonin also attenuates mitochondrial fragmentation and reduces apoptosis to protect VSMCs from calcification (130). On the other hand, in contrast to fission that exacerbates calcification, activation of Opa1-associated mitochondrial fusion can reverse this. In β-GP-induced calcification of VSMCs, activation of mitochondrial fusion and reduction of mitochondrial fission ameliorates VSMCs calcification (131).

As is the case in many cells, excessive mitochondrial fission generates large amounts of mitochondrial fragments that cause oxidative stress and disrupt normal mitochondrial respiration and ATP production. Thus, in addition to dysfunction, the calcification process is often accompanied by the reprogramming of mitochondrial energy metabolism due to low mitochondrial activity under pathological conditions (132, 133). Similar to the Warburg effect in cancer cells (134), mitochondria were found to decrease glucose oxidation and increase fatty acid oxidation during VSMC phenotypic transition (89). VSMCs may need to undergo this process during osteogenic differentiation and vascular calcification to obtain energy and produce the necessary biosynthetic precursors (135).

### Vascular fibroblasts (VAFs)

For a long time, endothelial cells and smooth muscle cells have received much attention as the major cellular components of the intima and interlayer of the vasculature, respectively. However, the adventitia has been considered solely as a structure that provides support and nutrition to the vasculature, and its role in vascular remodeling has been largely overlooked. The vascular adventitia is composed of a variety of cells, nerves, and connective tissue, with the most abundant cell type being fibroblasts (136). There is increasing evidence that the vascular adventitia is a key regulator of vessel wall structure and function, and the contribution of adventitial fibroblasts (AFs) to vascular remodeling has received considerable attention (137, 138). The vascular adventitia exhibited epigenetic thickening, an increased number of fibroblasts, and phenotypic alteration of AFs to myofibroblasts (MFs) in vascular remodeling (139). The transformed MFs exhibit enhanced proliferative and migratory activities and the presence of α-smooth muscle actin (α-SMA) (140). Recent studies have suggested that the switch for this phenotypic transition in fibroblasts may be related to mitochondrial function (141, 142).

It has been shown that mitochondrial fission contributes to the phenotypic switching of AFs (141). In AngII-treated primary mouse AFs, calcium-regulated phosphatase (CN)-dependent dephosphorylation of Drp1 induced mitochondrial fission, which stimulated AFs proliferation, migration, and phenotypic switching. In contrast, inhibition of heat shock protein 90 (HSP90) ameliorated CN expression and subsequent Drp1 activation, which ultimately attenuated vascular AngII-induced vascular adventitia remodeling. This demonstrates the involvement of HSP90 as a molecular chaperone of CN in the mitochondrial kinetic mechanism of AFs phenotypic transformation. Consistent with *in vitro* experiments, in an animal model of AngII-induced vascular outer membrane remodeling, inhibitors of HSP90 significantly reduced mitochondrial fission mediated by the CN/Drp1 signaling pathway, which in turn ameliorated AFs phenotypic conversion, vessel wall thickening, and aortic outer membrane fibrosis. In a recent study, Drp1 phosphorylation as well as fragmented mitochondria were significantly increased in transforming growth factor-β1 (TGF-β1)-treated primary AFs, leading to proliferation, migration, and phenotypic transformation of AFs. Moreover, inhibition of Drp1 by gene silencing could prevent this effect (142). Mechanistically, *in vitro* experiments demonstrated that hydrogen sulfide (H_2_S), a gas signaling molecule that regulates various cardiovascular functions, can block mitochondrial fission and improve TGF-β1-induced phenotypic conversion of AFs to MFs by regulating Rho-associated protein kinase 1-dependent Drp1 phosphorylation. This study reveals a novel mechanism of AFs phenotypic transformation and extravascular membrane remodeling involving mitochondrial dynamics.

Interesting observations by Lemmons et al. suggest that fibroblasts exhibit distinct metabolic phenotypes under both resting and proliferating conditions (143). Even under resting conditions, fibroblasts exhibit high metabolic activity compared to other cells (144). Fibroblasts could rapidly respond to hypoxic stress, and the ability of fibroblasts to proliferate, differentiate, and migrate under hypoxic conditions appears to be unique. However, chronic hypoxia still leads to phenotypic changes in fibroblasts (145). Studies indicated that the pulmonary artery adventitia harbors activated fibroblasts (PH-Fibs) show the characteristics of hyperproliferation, antiapoptotic, and proinflammatory phenotype in experimental hypoxic pulmonary hypertension (PH) and human PH (146). In response to vascular injury, fibroblasts can respond to environmental demands by rapidly altering their metabolic network. Therefore, one study explored the state of mitochondrial metabolism in PH-fibroblasts (147). In PH-Fibs, it was observed that pyruvate dehydrogenase was significantly inhibited, leading to increased glycolysis, which is consistent with a Warburg-like phenotype. The pro-oxidative state was further enhanced by increased mitochondrial superoxide production due to less efficient ATP synthesis. In addition, mitochondrial bioenergetics was suppressed in PH-Fibs and mitochondrial fragmentation was increased. Most importantly, the activity of complex I was greatly reduced. Conversely, acute and chronic hypoxia-induced increases in glycolysis in vascular fibroblasts were not accompanied by a lack of complex I (147). This evidence suggests that in the mechanism of vascular remodeling in PH, fibroblast mitochondrial metabolism may be altered differently from the effects of hypoxia, which in turn drives unique changes in cellular behavior.

### Macrophages

During atherosclerosis, macrophages retain the ability to phagocytose modified LDL and progressively transform into lipid-rich foam cells, which amplify and exacerbate the disease. It is widely believed that monocyte/macrophage-mediated inflammation accelerates the process of vascular remodeling (91). In the vascular system, monocytes differentiate into a heterogeneous population of macrophages, including M1 macrophages associated with pro-inflammatory processes and M2 macrophages associated with regression and repair (148).

The function of these polarized macrophages depends on the reprogramming of metabolism, which is essential for energy homeostasis (149). Indeed, mitochondrial dynamics are important for macrophage polarization and effector functions and are controlled by metabolic changes (150). For example, mitochondrial organization shifts to a hyperperfused state from a tubular and filamentous appearance and presents increased OXPHOS during nutrient starvation. Conversely, in the presence of nutrient overload, mitochondria are also hyperperfused, but in this way, they undergo altered OXPHOS associated with glycolytic metabolic characteristics (5). A recent study highlighted that mitochondrial dynamics are also associated with activation states during macrophage polarization *in vitro* (151). M1 macrophages have smaller mitochondrial networks, shorter branch lengths, lower mitochondrial membrane potential, and higher mitochondrial fission rates. This is due to the activation of Drp1 causing mitochondrial fragmentation in cells with enhanced glycolysis to support pro-inflammatory function. In contrast, the mitochondria of M2 macrophages are organized in larger networks, have longer branch lengths, and show higher fusion rates. This feature leads to better ETC and OXPHOS efficiency, supporting their anti-inflammatory and repair properties (151).

A recent study showed that macrophage Drp1 regulates this selective differentiation of macrophages (91). Macrophage-selective Drp1-deficient mice were created and were subjected to femoral artery wire injury. Drp1 deficiency also attenuated macrophage proliferation and accumulation in the damaged arteries of mice compared to control mice with attenuated intimal thickening and negative remodeling after vascular injury. Morphologically, inflammatory macrophages (M1) induced mitochondrial fission, whereas non-inflammatory/repair macrophages (M2) induced mitochondrial fusion. *In vitro* experiments showed that Drp1 induced the expression of molecules associated with inflammatory macrophages, while inhibition or knockdown of Drp1 reduced mitochondrial reactive oxygen species and chemotactic activity in cultured macrophages (91). These results suggest that in acute vascular injury, macrophage Drp1 deficiency can lead to reduced accumulation of inflammatory macrophages into injured vessels. Interestingly, macrophage mitochondrial fission may play a protective role in advanced atherosclerosis (152). In macrophage-enriched mouse atherosclerotic lesion areas, Drp1 levels were downregulated and Mfn2 levels were upregulated as the lesion progressed. A study conducted by Wang et al. demonstrated that Drp1-mediated mitochondrial fission has an important role in efferocytosis in atherosclerotic lesions in hyperlipidemic mice. Impaired efferocytosis induces the formation of large necrotic cores and leads to plaque vulnerability. This suggests that long-term blockade of Drp1 may be detrimental to cardiovascular events (153). Thus, how the delicate balance of mitochondrial fission and fusion regulates the role of macrophages in vascular remodeling is a question worth investigating.

Furthermore, co-culture experiments with macrophages and VSMCs showed that deletion of Drp1 in macrophages reduced macrophage-derived soluble factors, which in turn inhibited VSMCs growth and migration. This suggests that mitochondrial division inhibition may slow the process of atherosclerosis and vascular remodeling by disrupting the interaction between VSMCs and macrophages (91, 154).

## Mitochondrial dynamics and target-organ damage of vascular disease

### Myocardial hypertrophy and heart failure

Atherosclerosis is the pathological basis of various cardiovascular diseases, ultimately leading to cardiac insufficiency (155). Mitochondrial dynamics imbalance is also known to have an important place in cardiac diseases such as cardiac hypertrophy and heart failure. In transverse aortic banding (TAB)-treated mouse hearts, a phosphorylation-mediated increase in the number and loss of function of Drp1 mitochondria was found. The inhibitor mdivi-1 reduced the TAB-induced hypertrophic response and prevented PE-induced hypertrophic growth in rat neonatal cardiomyocytes (156). Treatment of neonatal rat cardiomyocytes with norepinephrine, a hypertrophic agonist, promotes mitochondrial fission characterized by a decrease in mean volume and an increase in relative number and leads to a decrease in mitochondrial function. Inhibition of Drp1 not only prevented mitochondrial fission but also blocked the hypertrophic growth of cardiomyocytes in response to norepinephrine. In the same study, inhibition of the fusion protein Mfn2 was also found to increase mitochondrial fission and stimulate the hypertrophic response (157). In addition, a reduced ratio of Mfn2 to Drp1 was observed in heart failure, while Mdivi-1 treatment normalized this ratio and thus improved cardiac function (158). Reduced expression of the fusion proteins Mfn2 and Opa1 was also observed *in vitro* in hypertrophic cardiomyocytes and a rat model of heart failure (159, 160). Interestingly, in a rat model of hypertension with cardiac insufficiency, promoting glucagon-like peptide-1 (GLP-1) production enhanced mitochondrial fusion in the heart and improved cardiac function (161). In a recent study, the oligopeptide Szeto-Schiller compound 31 (SS31) was found to significantly improve cardiac function, reduce myocardial interstitial fibrosis, and increase expression of optic atrophy-associated protein 1 (Opa1) in mice with pressure-overload heart failure by modulating mitochondrial fusion (162).

It is generally accepted that fused mitochondria are more efficient than fragmented mitochondria in the production of ATP and also compensate for the long-term accumulation of mitochondrial mutations (163). Therefore, many studies have been carried out based on the presence of mitochondrial fission or sensitivity to Drp1 chemical inhibitors, including Mdivi-1. Excessive mitochondrial fission has been proposed as a potential mechanism of cardiac dysfunction. Although dysregulated activation of Drp1 in response to stress may be detrimental to the heart, sustained inhibition of Drp1 is also detrimental because it eliminates the homeostatic function of Drp1 (164). Sustained inhibition of Drp1 through genetic deletion or repeated application of Mdivi-1 in the heart has been shown to exacerbate myocardial I/R injury (110). Taken together, it can be concluded that the balance between mitochondrial fusion and fission is a key factor in the onset and progression of myocardial hypertrophy and heart failure. Since Drp1 may have a fission-independent function (165), and given that inhibitors of Drp1 also appear to have some Drp1-independent effects (166), further studies are needed to clarify whether cardiac insufficiency is predominantly induced by excessive fission and whether fission inhibitors, such as Mdivi-1, can be used clinically for the treatment of cardiac disease.

### Ischemic stroke

Indeed, mitochondrial dynamics also play an important role in cell death and survival during cerebral ischemia. In the mouse middle cerebral artery occlusion (MCAO) I/R model, the emergence of mitochondrial fission was found to precede neuronal injury and neuronal cell death. Notably, NO-induced neuronal cell death could be alleviated by Mfn1 and dominant-negative Drp1, whereas overexpression of Drp1 or Fis1 caused cleavage and increased neuronal loss. Thus, sustained mitochondrial fission may play a causal role in NO-mediated neurotoxicity (167). Similarly, when Mdivi-1 was given as an inhibitor of Drp1 prior to cerebral ischemia, it attenuated ischemia-induced brain injury, reduced infarct volume, and improved neurological function. When given after ischemia and before reperfusion, Mdivi-1 also prevents brain injury by inhibiting mitochondrial fission-induced mitochondria-mediated apoptosis (168). A recent systematic review analyzed the effects of Mdivi-1 on mitochondria-mediated apoptosis and mitochondrial dysfunction in neuronal cells in the ischemia/reperfusion (I/R) injury after stroke model. The results suggest that Mdivi-1 protects mitochondrial function in neuronal cells by attenuating mitochondrial division, thereby attenuating I/R-induced brain injury and apoptosis (169). However, the specificity and efficacy of the pharmacological Drp1 inhibitor used, Mdivi-1, has now been questioned. A new study provides new possible mechanisms for the role of mitochondrial fission in neuronal injury (170). Deregulation of Drp1 repression by knocking down the mitochondrial A-kinase anchoring protein 1 (AKAP1) leads to increased stroke injury in mice. The evidence suggests that AKAP1 may protect against cerebral ischemic stroke by inhibiting Drp1-dependent mitochondrial fission, providing a new idea to resolve the current controversy about the role of mitochondrial fission in neuronal injury. Furthermore, Mfn2 was also found to be downregulated in the permanent MCAO model (171). Considering these evidences, increased mitochondrial fission should be considered an important factor contributing to ischemic stroke and brain injury.

### Renal injury

**FIGURE 3:**
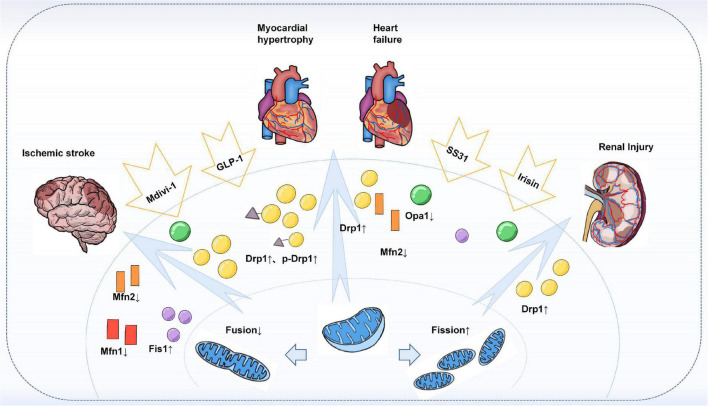
Mitochondrial dynamics in target-organ injury. Increased Drp1 and phosphorylated Drp1 were observed in an animal or *in vitro* models of cardiac hypertrophy, heart failure, stroke, and acute and chronic types of kidney injury, leading to enhanced mitochondrial division and increased fragmented mitochondria. At the same time, the expression of fusion proteins Mfns, especially Mfn2, and Opa1 was found to be reduced. Treatment with Mdivi-1, an inhibitor of Drp1, restored the Drp1 to Mfn2 ratio and improved cardiac function, attenuated brain injury, and reduced apoptosis in the kidney. In addition, others such as GLP-1, SS31, and Irisin have also been found to have a role in regulating mitochondrial dynamics, thereby reducing target organ damage.

Similar to organs such as the arteries, heart, and brain, a series of studies have identified mitochondrial dynamics in kidney injury. CVD is a major cause of mortality and morbidity in patients with chronic kidney disease (CKD), and vascular calcification (VC) is an independent predictor of cardiovascular mortality in CKD (172). A recent study found that in a chronic kidney disease model, Irisin restored mitochondrial function and reduced fission ameliorated CKD-associated vascular calcification, resulting in reduced serum creatinine, urea, and phosphorus levels in CKD mice. Mechanistically, Irisin restored mitochondrial function and alleviated mitochondrial fission *via* the AMPK/Drp1 signaling pathway, which further reduced CKD-associated vascular calcification (173). In addition, Drp1-induced mitochondrial fragmentation was observed in experimental models of renal ischemia/reperfusion and cisplatin-induced nephrotoxicity, demonstrating altered mitochondrial dynamics during acute kidney injury. Moreover, both acute kidney injury and apoptosis could be attenuated by the Drp1 inhibitor Mdivi-1 (174). Similar results were shown in a model of ischemic acute kidney injury (175). It was found that inhibition of Drp1-mediated mitochondrial fission plays an important role in ischemic preconditioning (IPC)-mediated renal protection (Figure 3).

## Conclusion remarks

In this review, we discussed recent advances in mitochondrial fusion, fission, and the delicate balance between them, focusing on the role of mitochondrial dynamics in several cells that make up the vascular wall in the involvement of vascular remodeling. We also discuss the function of mitochondrial fusion and division in target-organs of cardiovascular diseases. Related studies and applications of mitochondrial dynamics regulators, such as Mdivi-1, are also reviewed.

The dynamic balance of mitochondrial fission-fusion is the basis for maintaining normal mitochondrial morphology, number, distribution as well as the functional integrity of mitochondria. In mammalian cells, mitochondrial fusion is mainly controlled by Mfn1/2 and Opa1; while mitochondrial fission is controlled by Drp1, Fis1, Mff, and MiD49/51. However, this is not absolute, because some components regulating fusion, such as S-Opa1 and cardiolipin, are also involved in the mitochondrial division mechanism. In addition, MIEF1/2 is also involved in both fission and fusion. Recent findings also suggest that cells in different states my have different ways of mitochondrial division, which would also lead to different fate directions of the cells. Furthermore, the absence of either mitochondrial division or fusion alone can lead to abnormal cellular function, while division and fusion are also regulated by mitochondria and show a tendency to compensate for each other, suggesting that the delicate balance between division and fusion may be more important than the processes of division or fusion alone.

The typical vessel wall has a three-layer structure. The innermost layer consists of a tightly connected layer of endothelial cells (ECs), the endothelium, which is in direct contact with blood flow. The middle layer is mainly composed of smooth muscle cells (VSMCs) and elastic fibrous tissue. Fibroblasts, together with nerves and collagen, make up the outer membrane of the artery. The major cells in the three structural layers of the vessel wall all involved in vascular remodeling and are regulated by mitochondrial dynamics. Alterations in endothelial function are often considered to be the beginning of arterial remodeling, and mitochondrial division and fusion in ECs have important roles in regulating apoptosis, angiogenesis, and proinflammatory states. VSMCs have significant phenotypic plasticity, transforming to a synthetic phenotype after the loss of contractile markers, exhibiting dedifferentiation and increased proliferation and migration. Mitochondrial dynamics are involved in the regulation of this transition process. In addition, an imbalance of mitochondrial fusion and division may lead to VSMCs calcification, which is one of the important influences of vascular remodeling. The role of the arterial outer membrane and its major component, fibroblasts, in vascular remodeling has long been overlooked. There is increasing evidence that phenotypic changes from extravascular fibroblasts (AFs) to myofibroblasts (MFs) are important for the vascular remodeling process. Although there are still relatively few relevant studies, it is well-established that this phenotypic transition process is regulated by mitochondrial dynamics. Interestingly, how the delicate balance between mitochondrial fission and fusion regulates the role of macrophages in vascular remodeling is also worthy of further study.

The pathogenesis of cardiovascular disease, a leading cause of morbidity and mortality in developing countries, has been widely studied and focused on. Vascular remodeling associated with an imbalance in mitochondrial dynamics is an important player in several cardiovascular diseases. The heart, brain, and kidneys are all potential targets of vascular remodeling. Excessive mitochondrial division or reduced mitochondrial fusion has been found in diseases such as atherosclerosis, cardiac insufficiency, ischemic stroke, as well as acute and chronic kidney injury. In contrast, restoration of mitochondrial dynamics homeostasis by silencing related genes, using division inhibitors or fusion promoters may provide some protection. This suggests that inhibiting mitochondrial division or promoting mitochondrial fusion to maintaining mitochondrial kinetic homeostasis is promising new targets for the treatment of vascular remodeling and its associated cardiovascular diseases. However, how to finely regulate the activity of mitochondrial dynamics proteins while reducing the possible side effects is one of the main challenges at hand.

Since Cassidy-Stone et al. reported in 2008 that Mdivi-1 inhibits excessive mitochondrial fission and enhances mitochondrial fusion activity, which can protect cells from toxic injury, a large number of studies on the regulation of mitochondrial dynamics have emerged. In the last decades, some progress has been made in the development of mitochondrial fission inhibitors such as Mdivi-1, P110 and Dynasore. Inhibitors of mitochondrial fission may be promising therapeutic targets in neurodegenerative disease disorders such as Alzheimer’s disease (AD) and Huntington’s disease (HD). However, no drug formulations have been reported that are available for use in humans. In mechanistic studies of cardiovascular disease, beneficial effects of mitochondrial dynamic modulators have been reported at both the cellular and animal levels. Overall, mitochondrial kinetic modulators have good research prospects for the treatment of human diseases and may provide new therapeutic approaches for cardiovascular diseases in the future. Considering the differences in mitochondrial energy requirements and functions in different cell types, especially between cardiomyocytes and other cells, selective modulation of mitochondrial dynamics in cells is also worth considering. Therefore, whether the mitochondrial dynamics modulators used in basic experiments, such as Mdivi-1, an inhibitor of Drp1, and P110, an adapter-specific inhibitor of Drp1/Fis1 interaction discovered in recent years, can be used for the treatment of related cardiovascular diseases still needs to be verified by numerous clinical studies, and their related toxicological and pharmacokinetic characteristics still need to be further investigated. This will be a new direction for future research.

## Author contributions

TZ, JQ, and JZ structured the manuscript and contributed to the figures. QH, YY, and HY revised the manuscript and confirmed the final revision. All authors contributed to the manuscript preparation and approved the submitted version.
